# Frequency of Thunder Storms

**Published:** 1892-06

**Authors:** 


### FREQUENCY OF THUNDER STORMS.

A German periodical gives statistics concerning the frequency of thunder storms in various regions of the world. Java has thunder storms on the average 97 days in the year; Sumatra, 86; Hindoostan, 56; Borneo, 54; the Gold Coast, 52; Rio de Janeiro, 51; Italy, 38; West Indies, 36; South Guinea, 32; Buenos Ayres, Canada, and Austria, 23; Baden, Wurtemberg and Hungary, 22; Silesia, Bavaria, and Belgium, 21; Holland, 18; Saxony and Bradenburg, 17; France, Austria and South Russia, 16; Spain and Portugal, 15; Sweden and Finland, 8; England and the high Swiss mountains, 7; Norway, 4; Cairo, 3. In East Turkestan, as well as in the extreme north, there are almost no thunder storms. The northern limits of the thunder storms are Cape Ogle, northern part of North America, Iceland, Novaja, Semelja, and the coast of the Siberian ice sea.

